# Atmospheric Semi- and Intermediate-Volatility Organic Compounds: Recent Progress and Future Perspectives

**DOI:** 10.3390/toxics14100925

**Published:** 2026-10-19

**Authors:** Zichen Ma, Yuanyuan Ji, Chunmei Geng, Hong Li, Xiaoli Wang, Wen Yang

**Affiliations:** 1 State Key Laboratory of Environmental Criteria and Risk Assessment, Chinese Research Academy of Environmental Sciences, Beijing 100012, China; 2 School of Environmental Science and Engineering, Tianjin University of Technology, Tianjin 300384, China

**Keywords:** semi-volatile organic compounds, intermediate-volatility organic compounds, pollution characteristics, secondary organic aerosol, health risks

## Abstract

Semi-volatile and intermediate-volatility organic compounds (S/IVOCs) represent a chemically complex class of atmospheric organics that contribute substantially to secondary organic aerosol (SOA) formation and may pose health risks. This review synthesizes recent advances in S/IVOC measurement, ambient concentrations and composition, spatiotemporal variations, SOA formation, and health effects. Online techniques provide high-time-resolution measurements, whereas offline GC–MS and GC×GC–MS offer superior capabilities for compound separation and identification. However, unresolved complex mixtures, sampling artifacts, limited standards, and variable instrumental responses remain major challenges. Field observations reveal pronounced differences in S/IVOC levels and composition among industrial, urban, traffic-influenced, and background environments, while seasonal and diurnal patterns vary across sites. Evidence from field observations, source-emission studies, chamber experiments, and model simulations indicates that S/IVOCs are important SOA precursors, although quantitative estimates remain subject to considerable uncertainties arising from precursor coverage, yield parameters, oxidation conditions, gas–particle partitioning, emission inventories, and model parameterizations. Current health evidence is largely confined to compounds with established toxicity data, particularly polycyclic aromatic hydrocarbons, whereas unresolved components and oxidation products remain poorly characterized. Overall, our synthesis suggests that the key challenge in current S/IVOC research is to reduce uncertainties across measurement, ambient characterization, SOA contribution, and health-risk assessment through standardized measurements, coordinated long-term observations, improved emission and aging representations, and broader toxicological characterization.

## 1. Introduction

Secondary organic aerosol (SOA), formed through the oxidation of precursors such as volatile organic compounds (VOCs), constitutes a significant chemical component of PM_2.5_ and can account for up to 80% of organic aerosol (OA), with important implications for atmospheric visibility, environmental quality, climate, and human health [1,2,3]. For decades, atmospheric chemical transport models based on conventional emission inventories and mechanisms have substantially underestimated SOA relative to field observations, leaving a persistent gap in the atmospheric OA budget [4]. In 2007, Robinson et al. [4] first proposed that conventional studies had predominantly focused on more volatile VOCs, largely overlooking the primary emissions and subsequent oxidation of intermediate-volatility organic compounds (IVOCs) and semi-volatile organic compounds (SVOCs). These compounds, with lower saturation vapor pressures, likely contribute significantly to the observed SOA underestimation in models.

According to the volatility basis set (VBS) framework proposed by Donahue et al. [5], organic compounds can be classified according to their effective saturation concentration (C*). In this review, IVOCs are thermodynamically defined as compounds with C* values of 10^3^–10^6^ μg m^−3^, whereas SVOCs have C* values of approximately 10^−1^–10^3^ μg m^−3^ [5,6,7,8]. The term S/IVOCs is used collectively to refer to these two volatility classes. For hydrocarbons, these volatility ranges correspond approximately to C12–C22 n-alkanes for IVOCs and C22–C32 n-alkanes for SVOCs [6,7,8]. These carbon-number ranges are used only as approximate reference ranges for n-alkanes rather than universal structural definitions, because molecular functionality, branching, cyclic structure, and heteroatom content can substantially alter vapor pressure and effective saturation concentration. In addition, individual field and laboratory studies may operationally define the measured S/IVOC fraction according to chromatographic retention windows, sampling phase, and analytical coverage. Therefore, operationally measured S/IVOC fractions are not necessarily equivalent among studies and are specified where relevant in this review. Although the mass concentrations of S/IVOCs are generally lower than those of conventional VOCs, their importance for SOA formation can be disproportionate under specific conditions. During the CalNex campaign in Pasadena, primary IVOCs accounted for only 7.4 ± 1.2% of the concentration of speciated VOCs but were estimated to produce approximately 30% of newly formed afternoon SOA, about five times the contribution from single-ring aromatics [8]. In high-NOx smog-chamber experiments, Tkacik et al. [9] estimated that UCM vapors from diesel exhaust had an SOA formation potential nearly four times that of single-ring aromatics after several hours of oxidation under high-NOx chamber conditions.

Early studies of S/IVOCs focused mainly on motor vehicle exhaust and fossil-fuel combustion. Zhao et al. [10] demonstrated that diesel exhaust contains substantial hydrocarbon material unresolved by conventional chromatography, referred to as an unresolved complex mixture (UCM), and identified the IVOC fraction as an important precursor of urban SOA. As transportation-related VOC emissions have declined under increasingly stringent emission controls, the relative importance of other urban organic emission sources has increased. In addition to combustion-related emissions, the use of volatile chemical products (VCPs) is an important source of urban organic emissions. McDonald et al. [11] investigated emissions from products such as pesticides, coatings, printing inks, adhesives, cleaning agents, and personal care products. Organic solvents used in these products are largely derived from petrochemical feedstocks and can release both IVOCs and higher-volatility VOCs through evaporation during normal product use. Beyond urban anthropogenic sources, biomass burning represents another major source of S/IVOCs. Li et al. [12] used a laminar-flow oxidation reactor to investigate SOA formation from wood-burning emissions and estimated that IVOCs contributed approximately 70% of the SOA formed in the reactor, more than twice the contribution attributed to VOCs.

Despite growing recognition of the importance of S/IVOCs in atmospheric chemistry, field observations remain relatively limited because of their complex chemical composition, numerous isomers, and susceptibility to sampling losses. Moreover, considerable differences exist among sampling and analytical methods used in different studies, such as offline GC–MS and online PTR–MS [13,14].

Previous reviews have addressed different aspects of S/IVOC research. Tan et al. [15] summarized the major measurement techniques for atmospheric S/IVOCs, with particular emphasis on gas chromatography- and chemical ionization mass spectrometry-based methods. Tang et al. [16] reviewed the measurement techniques, sources, atmospheric behavior, and contributions of S/IVOCs to organic aerosol formation. Wang et al. [17] provided a broader overview of IVOCs, including their definitions, measurement techniques, field observations, laboratory mechanisms, and model simulations. Ling et al. [18] focused primarily on the roles of S/IVOCs in SOA formation, particularly their representation in emission inventories and atmospheric models. More recently, Yan et al. [19] reviewed the pollution characteristics and environmental impacts of atmospheric IVOCs, including their potential health risks. Nevertheless, these previous reviews generally focused on specific aspects of S/IVOCs or primarily on IVOCs, and an integrated assessment linking analytical methods, ambient concentrations and composition, SOA formation, and health-risk evaluation remains limited. Therefore, the present review aims to provide an integrated synthesis linking analytical methods, ambient characterization, SOA assessment, and health-risk evidence, with particular emphasis on how uncertainties originating from measurement and compound coverage propagate into subsequent estimates of atmospheric abundance, SOA formation, and health-risk characterization.

The literature reviewed in this study was identified primarily through searches of Google Scholar, Web of Science, and the China National Knowledge Infrastructure (CNKI). Publications from 1996 to 2026 were considered. The principal search terms included “S/IVOCs”, “semi-volatile organic compounds”, “intermediate-volatility organic compounds”, “field observations”, “measurement”, “measurement techniques”, “secondary organic aerosol”, “SOA”, and “health risks”, together with corresponding Chinese terms where applicable. No restrictions were imposed on journal titles or geographical regions. The final literature search was conducted on 1 September 2026. More than 113 publications were screened for relevance, of which approximately 60 studies were retained as key references for the present review. Studies were included when they directly addressed atmospheric S/IVOCs, particularly field measurements, analytical methods, ambient concentrations and composition, SOA formation, emission characteristics, or health-related evidence relevant to atmospheric exposure. Studies focused primarily on indoor S/IVOC measurements were generally excluded. However, indoor studies relevant to the health effects of S/IVOCs were also retained. Modeling studies without a clear connection to atmospheric observations or to the interpretation of field measurements were also generally excluded, whereas representative modeling studies relevant to SOA formation and atmospheric processes were retained where necessary. For studies with substantially overlapping datasets or research content, priority was given to the most recent and/or most comprehensive publication.

Based on this literature search, this article compares major analytical approaches for S/IVOCs, summarizes their concentration and compositional characteristics across different environments, discusses their contributions to SOA formation, and evaluates their potential human health risks. Through this synthesis, we argue that the major limitation in current S/IVOC research lies in the substantial uncertainties that connect measurement, ambient characterization, SOA formation, and health-risk assessment, and that future progress requires a more integrated framework combining standardized measurements, coordinated long-term observations, improved representation of emissions and atmospheric aging, and expanded toxicological characterization.

## 2. Sampling and Analytical Methods for S/IVOCs

Current sampling and analytical methods for S/IVOCs can be broadly grouped into online and offline approaches according to the sampling–measurement configuration [20]. Online approaches generally integrate sampling and analysis in real time or near real time at the field site, whereas offline approaches involve separate sample collection followed by subsequent laboratory pretreatment and analysis [20]. Within both configurations, different analytical techniques, including chromatographic and mass spectrometric methods, may be employed.

### 2.1. Online Methods

Table 1 summarizes key developments in online analytical methods for S/IVOCs. Online chromatographic techniques integrate automated sampling and thermal-desorption modules to achieve species-level characterization. As early as the 1990s, in situ automated GC–MS was used for VOC analysis at remote background sites. For example, Helmig et al. [21] developed a ppt-level multisorbent preconcentration and thermal-desorption method enabling measurements of compounds up to C_12_–C_15_, including some S/IVOC species. To extend the accessible volatility range, Williams et al. [22] used a thermal desorption aerosol gas chromatograph (TAG) to measure more than 300 semi-volatile and nonvolatile particle-phase organic compounds at hourly resolution. Building on TAG, the semi-volatile thermal desorption aerosol gas chromatograph (SV-TAG) further extended sampling and analysis to both gas- and particle-phase S/IVOCs, enabling online species-resolved measurements at a temporal resolution of 1–2 h [14]. However, the temporal resolution remains at the hourly scale, and some thermally labile compounds may decompose during thermal desorption. Online thermal desorption GC–MS (online TD-GC–MS), widely used in routine observations, offers a high degree of automation and relatively stable quantification and has been applied to continuous automated measurements of atmospheric n-alkanes and polycyclic aromatic hydrocarbons (PAHs) [23]. To address the limited peak capacity of one-dimensional gas chromatography (1D-GC), which causes extensive isomer co-elution and UCM formation [24], comprehensive two-dimensional gas chromatography (GC×GC) coupled with time-of-flight mass spectrometry (ToF-MS) substantially increases peak capacity and improves the resolution of UCM components [15]. This approach expands the screening of atmospheric S/IVOCs and enables UCM components to be classified into chemical classes such as n-alkanes, branched alkanes, cycloalkanes, and aromatic hydrocarbons [20,24,25,26].

Among direct-ionization mass spectrometric techniques, high-resolution time-of-flight chemical ionization mass spectrometry (HR-ToF-CIMS) ionizes analytes through chemical reactions with reagent ions and enables high-time-resolution online measurements of atmospheric trace species. Its selectivity is reagent-ion dependent rather than inherently target-specific: the detectable compound range and instrumental sensitivity vary with the reagent ion and analyte chemistry [28]. Different reagent ions preferentially detect different compound classes, and the use of multiple reagent ions can broaden chemical coverage [28]. HR-ToF-CIMS has been applied to the measurement of highly oxygenated organic molecules (HOMs) and other polar oxidation products [27,28]. However, the resulting mass spectra mainly provide molecular-formula or mass-to-charge information, making isomer discrimination and quantitative characterization challenging. Extractive electrospray ionization time-of-flight mass spectrometry (EESI-ToF-MS) extracts aerosol particles into electrospray droplets for ionization, enabling nonthermal online analysis of particle-phase organics and avoiding possible thermal decomposition or fragmentation during conventional thermal-desorption introduction. EESI-ToF-MS can provide information at or near the molecular-formula level; however, assignment of a molecular formula does not establish molecular structure or isomer identity. Quantification also remains challenging because the instrumental response depends on the electrospray solution composition, ion polarity, and calibration conditions [28].

### 2.2. Offline Methods

Because of their strong capabilities for species identification, offline methods remain fundamental for S/IVOC compositional characterization and source-profile development [16]. These methods generally involve three stages: field sampling, laboratory pretreatment, and qualitative/quantitative analysis. Based on differences in sampling and pretreatment procedures, they can be divided into three main categories (Table 2).

The first category is filter–adsorbent sampling, typically using a quartz-fiber filter (QFF) in series with polyurethane foam (PUF) or XAD-2 resin to separately collect particle- and gas-phase S/IVOCs, with samples readily stored after collection [20,29]. Because S/IVOCs partition between gas and particle phases, sampling artifacts can introduce both positive and negative biases. For instance, adsorption of gas-phase S/IVOCs onto filters may cause positive bias, whereas re-volatilization of collected particle-phase compounds during prolonged sampling can lead to negative bias. Sorbent selection and capacity also affect the recovery of gas-phase S/IVOCs because different sorbents exhibit different retention characteristics and compound coverage [15,20]. For active sampling, excessively large sampled air volumes can cause breakthrough when the retention capacity of the sorbent is exceeded, resulting in underestimation of gas-phase concentrations [31]. Breakthrough depends on compound volatility, sorbent properties and volume, temperature, and total sampled air volume [31]. Because sampling flow rate and sampling duration together determine the total air volume collected, these parameters should be controlled in relation to sorbent capacity when comparing measurements among studies. During laboratory pretreatment and analysis, conventional solvent extraction is a well-established approach for qualitative and quantitative determination of alkanes, PAHs, phthalates and other common compounds [29,30]. After cleanup, extracts are analyzed by GC–MS. Although this method is mature and reliable, sample pretreatment is labor-intensive and consumes substantial amounts of organic solvents [26,30].

The second category uses thermal-desorption tubes packed with sorbents such as Tenax TA/GR to collect gas-phase S/IVOCs. After sampling, target compounds are thermally desorbed and introduced into a GC–MS or GC×GC–MS system. TD-GC–MS requires no organic solvents and avoids solvent-peak interference. Comparative studies have shown broadly consistent results between thermal desorption and solvent extraction for several compound classes, although differences may occur for relatively volatile, low-carbon SVOCs; thermal desorption can provide similar or lower detection limits for some of these compounds [15]. Therefore, analytical sensitivity should be regarded as analyte- and method-specific rather than as an inherent advantage of TD-GC–MS. In addition, both target compounds and sorbents must be sufficiently thermally stable to avoid decomposition at elevated temperatures [20]. Wang et al. [30] developed a TD-GC–MS method using an ionic-liquid-modified Tenax GR sorbent to determine 10 SVOCs in indoor air and verified the accuracy and stability.

The third category combines filter/adsorbent sampling with GC×GC–MS or GC×GC-ToF-MS non-target analysis for in-depth characterization of complex S/IVOCs and UCM components. Gas- and particle-phase samples are first collected using the corresponding sampling media and are subsequently introduced into GC×GC–MS/GC×GC-ToF-MS after solvent extraction or thermal desorption in the laboratory. Compared with one-dimensional GC–MS, GC×GC provides orthogonal separation using two columns with different separation mechanisms, substantially increasing peak capacity and chromatographic resolution. Coupling with ToF-MS further enhances the qualitative characterization of complex organic mixtures.

### 2.3. Limitations and Uncertainties of Current Analytical Methods

The distinction between online and offline approaches in this review refers primarily to the sampling–measurement configuration, particularly whether sampling and analysis are integrated at the field site or separated into field collection and subsequent laboratory analysis. Online methods offer high temporal resolution, but species identification is often less comprehensive than that achieved by offline GC×GC. Offline GC×GC provides superior resolving power but requires longer analytical times and more complex data processing. However, both approaches face challenges in fully characterizing the S/IVOC fraction targeted by a measurement because of incomplete compound coverage, sampling losses, unresolved components, and compound-dependent instrumental responses. Regardless of the sampling–measurement configuration, current analytical methods may not fully quantify the S/IVOC mass within their targeted volatility range and phase coverage. This limitation arises from incomplete compound coverage, sampling losses, unresolved components, and compound-dependent instrumental responses. Future studies should further integrate complementary online and offline approaches with high-resolution instrumentation to improve molecular-level quantification of gas- and particle-phase S/IVOCs.

## 3. Pollution Characteristics of S/IVOCs

### 3.1. Concentration Characteristics of S/IVOCs

Observed atmospheric S/IVOC concentrations exhibit large variability across different site types, sampling periods, and measurement approaches (Table 3).

A number of studies have observed elevated S/IVOC concentrations in near-source and industrial settings, with highly variable reported values. Hou et al. [32] conducted measurements in August 2024 and January 2025 at a riverside petrochemical industrial park in Yangzhou and four surrounding site types. Hou et al. [32] reported mean ambient IVOC concentrations (gas + particle) of 172 ± 25.0 μg m^−3^ in summer and 31.8 ± 2.45 μg m^−3^ in winter for the riverside industrial park. Zhang et al. [33] reported mean gas- and particle-phase IVOC concentrations of 108.87 ± 78.93 and 4.22 ± 1.54 μg m^−3^, respectively, in a petrochemical industrial area in summer 2021, indicating that near-source IVOCs were predominantly present in the gas phase during the study period. Measurements at Shanghai Yangshan Port in summer 2016 and winter 2017 showed that shipping activities in the port and adjacent waters substantially affected local IVOC pollution. The overall mean gas-phase IVOC concentration across the two seasonal campaigns was 5.1 ± 0.8 μg m^−3^ [34], with seasonal means of 5.7 ± 0.3 μg m^−3^ in summer and 4.6 ± 0.7 μg m^−3^ in winter.

In urban areas, reported mean S/IVOC concentrations generally range from several to several tens of μg m^−3^. Li et al. [35] conducted winter and summer observations during pollution episodes at an urban site in Shanghai and reported mean gas-plus-particle IVOC concentrations of 58.5 ± 27.0 and 6.8 ± 3.7 μg m^−3^, respectively. Li et al. [36] reported a mean gas-phase S/IVOC concentration of 9.87 ± 5.92 μg m^−3^ (2.4–20.7 μg m^−3^) in Shenzhen in winter 2023. Zhou et al. [24] measured S/IVOCs in Chengdu during winter 2021 and found mean gas- and particle-phase concentrations of 2.63 ± 1.25 and 1.83 ± 1.15 μg m^−3^, respectively, with higher levels in the gas phase. At an urban site in Guangzhou in autumn 2019, the mean gas-phase IVOC concentration was 25.0 ± 0.95 μg m^−3^ [37]. Numerically lower concentrations have been reported in several studies conducted outside China. For example, the mean IVOC concentration in Pasadena, Los Angeles, in summer 2010 was 6.3 ± 1.9 μg m^−3^ [8], whereas the total gas-plus-particle concentrations of aliphatic and aromatic S/IVOCs across four London sites during winter–spring 2017 ranged from 0.587 to 2.448 μg m^−3^ [25].

Lower reported values have also been observed in background and remote environments. During the 2014 wet and dry seasons in the Amazon forest, 30 sesquiterpenes and four diterpenes were observed at mean individual concentrations of 0.00001–0.00604 μg m^−3^; their summed concentrations were 0.0418 μg m^−3^ in the wet season and 0.0388 μg m^−3^ in the dry season [38]. In a Colorado coniferous forest in summer 2011, the total S/IVOC mass concentration was approximately 0.7 μg m^−3^ [14].

The reported S/IVOC concentrations should be compared with caution because the studies summarized in Table 3 differ in operational definitions, phase coverage, compound coverage, sampling and analytical methods, observation periods, and source environments. Therefore, the generally higher concentrations reported in source-influenced and industrial environments and the lower values observed at some remote and background sites should be interpreted as a qualitative tendency rather than a strict quantitative spatial gradient. Similarly, differences between Chinese and non-Chinese measurements cannot be attributed to geographical location alone without considering these methodological and environmental factors.

### 3.2. Compositional Characteristics of S/IVOCs

Atmospheric S/IVOCs comprise a highly complex mixture containing numerous structurally diverse compounds. With the application of high-resolution techniques such as GC×GC, portions of the UCM that previously appeared as co-eluting chromatographic envelopes can increasingly be resolved into specific chemical classes. Based on molecular structure and functional groups, resolvable atmospheric S/IVOCs can be broadly categorized into aliphatic hydrocarbons, aromatic hydrocarbons, and non-hydrocarbon compounds containing oxygen, nitrogen, or sulfur.

Aliphatic hydrocarbons are common and frequently investigated components of atmospheric S/IVOCs and mainly include n-alkanes, branched alkanes, and cycloalkanes. In a study of traffic-related S/IVOCs in London, Xu et al. [25] identified C_13_–C_36_ linear and branched alkanes, C_12_–C_25_ monocyclic alkanes, and C_13_–C_27_ bicyclic alkanes, indicating that structurally diverse aliphatic hydrocarbons are important components of S/IVOCs in traffic-influenced environments. Because branched and cyclic hydrocarbons comprise numerous isomers, they often appear as part of the UCM in conventional one-dimensional chromatographic analysis. Using GC×GC, Xu et al. [25] further separated monocyclic and bicyclic alkanes from the UCM; these groups accounted for 17–24% and 8–15%, respectively, of the total identified and quantified S/IVOC mass across the combined gas- and particle-phase samples. Zhao et al. [10] also reported that more than 90% of IVOCs in diesel-vehicle emissions occurred as UCM, indicating that a substantial fraction of complex traffic-related components remains unresolved at the molecular level. Aliphatic hydrocarbons are therefore an important component of traffic-related S/IVOCs, and their complex branched and cyclic structures remain a key focus of current UCM characterization.

Aromatic components mainly include monocyclic aromatic hydrocarbons, PAHs, and their derivatives, such as naphthalene, fluorene, phenanthrene, anthracene, pyrene, fluoranthene, methylnaphthalenes, and biphenyl. Although aromatic compounds often represent a relatively small fraction of the measured S/IVOC mass, their chemical reactivity, SOA formation potential, and toxicity make them an important group of S/IVOCs. Xu et al. [25] identified C_10_–C_24_ monocyclic aromatic hydrocarbons as one of the major S/IVOC groups in traffic-influenced London air and also detected PAHs including naphthalene, fluorene, phenanthrene, anthracene, pyrene, and fluoranthene. Fang et al. [37] reported that naphthalene was the most abundant gas-phase PAH in urban Guangzhou, accounting for 67.9% ± 6.8% of the measured PAHs. Aromatic hydrocarbons such as naphthalene and methylnaphthalenes were also well correlated with combustion tracers such as acetylene, suggesting that combustion may be an important source of primary hydrocarbon IVOCs in urban air. Zhang et al. [33] observed phase-dependent PAH distributions in a petrochemical industrial area, with gas-phase PAHs dominated by naphthalene and phenanthrene and particle-phase PAHs dominated by fluorene. These findings indicate that the concentration and composition of aromatic S/IVOCs are influenced by both emission sources and volatility.

In addition to hydrocarbons, oxygenated and other heteroatom-containing compounds represent an important but methodologically challenging fraction of atmospheric S/IVOCs. Oxygenated S/IVOCs can arise from both primary emissions and secondary atmospheric oxidation, and these two origins should be distinguished. Li et al. [36] found that acids were the most abundant group in gas-phase S/IVOCs in Shenzhen during winter, accounting for 41.6%, followed by ketones and oxygenated PAHs at 13.6% and 8.3%, respectively, all exceeding the alkane fraction of 7.6%. Non-target analysis of wintertime S/IVOCs in Chengdu by Zhou et al. [24] showed that gas-phase S/IVOCs were dominated by acids and ketones, whereas alcohols and esters predominated in the particle phase. VCP-related tracers such as Texanol were also detected. These ambient observations, however, do not by themselves establish whether individual oxygenated compounds are emitted directly or formed secondarily in the atmosphere. Direct source measurements provide clearer evidence for primary oxygenated IVOCs. Wang et al. [39] identified oxygenated IVOCs directly in evaporative emissions from in-use gasoline vehicles, including aldehydes, ethers, esters, ketones, alcohols, phenols, and quinones. In contrast, oxygenated S/IVOCs may also be produced through atmospheric oxidation of less-oxygenated precursors. Yee et al. [38] observed sesquiterpenes together with their oxidation products in the central Amazon and identified oxidation products in both gas and particle phases, providing direct evidence for secondary formation of lower-volatility oxygenated organics.

The reported abundance and classification of oxygenated S/IVOCs are also strongly method-dependent. Chromatographic techniques such as GC×GC–MS can separate compounds according to volatility and polarity and, when authentic standards or reliable mass spectra are available, provide structural information for individual oxygenated compounds [39]. Derivatization-based SV-TAG analysis can extend chromatographic measurements to more highly oxygenated species [38]. In contrast, soft-ionization high-resolution mass spectrometric techniques such as CIMS and EESI can provide broader or higher-time-resolution coverage of polar oxidation products, but their detectable compound range depends on ionization chemistry and the resulting molecular-formula information does not necessarily establish molecular structure or isomer identity [28]. Consequently, “oxygenated S/IVOCs” reported by different studies may represent different subsets of compounds, and direct quantitative comparison requires consideration of analytical coverage and operational definitions.

### 3.3. Spatiotemporal Variations in S/IVOCs

Gas-phase S/IVOC concentrations can vary markedly over different temporal scales, including diurnal and seasonal scales, under the combined influence of emission intensity, meteorological conditions, and photochemical processing.

#### 3.3.1. Evidence from Ambient Measurements

Meteorological conditions are important considerations when interpreting the temporal variability in S/IVOCs. In the studies discussed below, information on wind direction, air-mass transport, atmospheric mixing, and temperature was used to help explain observed seasonal and diurnal variations [32,34,40]. However, such analyses should be distinguished from meteorological normalization, which requires explicit adjustment of measured concentrations for meteorological influences. Therefore, the reported temporal variations should not be interpreted as changes independent of meteorological conditions.

Seasonal patterns of S/IVOCs are not consistent across studies. Li et al. [35] compared winter and summer pollution episodes at the same urban site in Shanghai and found a mean atmospheric IVOC concentration of 58.5 ± 27.0 μg m^−3^ in winter, substantially higher than the summer value of 6.8 ± 3.7 μg m^−3^. This seasonal difference may reflect combined effects of meteorology, pollution type, photochemical activity, and source profiles; enhanced summertime photochemistry may also deplete certain highly reactive IVOCs. In contrast, Wang et al. [34] reported an overall mean IVOC concentration of 5.1 ± 0.8 μg m^−3^ across the summer and winter campaigns at Shanghai Yangshan Port, with season-specific mean concentrations of 5.7 ± 0.3 μg m^−3^ in summer and 4.6 ± 0.7 μg m^−3^ in winter. The authors noted that southerly and southeasterly airflows during the summer campaign more readily transported ship emissions from the port area to the observation site, whereas winter air masses more often originated from northern continental regions. Differences in seasonal sources, shipping activity, and meteorology may therefore explain the seasonal pattern that differed from that observed in urban Shanghai. Hou et al. [32] reported a much stronger summer-high/winter-low pattern in a riverside petrochemical industrial park in Yangzhou, with mean ambient IVOC concentrations of 172 ± 25.0 μg m^−3^ in summer and 31.8 ± 2.45 μg m^−3^ in winter. Higher summer temperatures enhanced evaporation of petroleum and chemical products, while higher water levels and more intensive navigation increased inland shipping emissions; lower winter temperatures suppressed industrial evaporation and coincided with reduced shipping activity, producing the pronounced seasonal difference.

Diurnal variations in S/IVOCs are likewise affected by emission sources, volatility, and atmospheric chemistry. Hou et al. [32] conducted measurements in a petrochemical industrial park in Yangzhou, Jiangsu, during 16–23 August 2024 and 2–9 January 2025. In both summer and winter, IVOC concentrations were higher in the morning, decreased in the afternoon, and reached their lowest levels in the evening, which the authors attributed to a combination of morning traffic and industrial emissions, changes in atmospheric mixing, and enhanced afternoon photochemical processing; by comparison, intraday differences in chemical composition were limited. Fang et al. [37] observed urban Guangzhou from 23 September to 4 October 2019 and found that primary hydrocarbon IVOCs were relatively high in the morning and evening, whereas total IVOCs increased at 12:00–15:00, suggesting that midday formation of secondary oxygenated UCM components may alter the diurnal pattern of total IVOCs. For SVOCs, Wernis et al. [40] reported that temperature-dependent components increased mainly from noon to afternoon at suburban Livermore, California, during 11–21 April 2018, whereas biomass-burning-related components increased primarily at night. Measurements by Chan et al. [14] at the Manitou Experimental Forest Observatory in the Rocky Mountains, Colorado, during 19–30 August 2011 likewise showed higher nighttime levels of biogenic components within S/IVOCs, whereas components dominated by anthropogenic species such as PAHs were higher during the daytime.

#### 3.3.2. Evidence from Emission Inventories

Xu et al. [41] developed a global anthropogenic full-volatility-range organic compound (FVOC) emission inventory for 1970–2020 using the MEIC-global-FVOC framework. In this inventory, FVOCs comprise LVOCs, SVOCs, IVOCs, and NMVOCs. The estimated global SVOC emissions increased from 8.7 Mt in 1970 to 13.5 Mt in 2020, while IVOC emissions increased from 20.7 to 35.7 Mt. Relative to total FVOC emissions, the SVOC fraction remained approximately 8.2%, whereas the IVOC fraction increased from 19.5% to 21.6% [41]. Within the MEIC-global-FVOC framework, driving-factor decomposition indicated that the increases in L/SVOC and IVOC emissions were predominantly associated with growth in activity rates, while changes in technology distributions, emission factors, and emission controls had smaller effects on the long-term trend. Nevertheless, these values should be interpreted as inventory-based estimates rather than direct observations. Their absolute magnitudes and relative contributions remain subject to uncertainties in source-specific emission factors, regional representativeness, emission-control assumptions, and source coverage [41].

At the regional scale, Wu et al. [42] first developed an S/IVOC emission inventory for the Pearl River Delta (PRD) for 2010, estimating total regional emissions of 323.4 Gg. On-road mobile and industrial sources were the major contributors, and high-emission areas were mainly concentrated in central cities such as Guangzhou, Foshan, Dongguan, and Shenzhen. Subsequently, Wu et al. [43] developed a gridded S/IVOC emission inventory for China for 2016 using updated source-specific emission parameters and broader source coverage. High emissions were mainly distributed in highly industrialized and urbanized regions, including the Beijing–Tianjin–Hebei (BTH), Yangtze River Delta (YRD), Pearl River Delta (PRD), and Sichuan–Chongqing (SC) regions. Among these four major urban agglomerations, total S/IVOC emissions were the highest in the YRD, followed by BTH, SC, and the PRD. Overall, existing inventories indicate pronounced regional clustering of S/IVOC emissions in China, broadly associated with the spatial distributions of industrial, residential, and transportation sources.

### 3.4. Limitations and Uncertainties in Current Studies of Pollution Characteristics

Current field observations of S/IVOCs remain limited in both temporal coverage and spatial representativeness. Most field studies rely on short-term campaigns lasting several weeks to several months and lack long-term continuous observations. Seasonal and diurnal variations in S/IVOCs are jointly controlled by emissions, meteorology, and photochemical processes, and the observed patterns differ among regions and seasons. Short-term results can therefore be strongly affected by episodic meteorological conditions or transient emission events and may not capture long-term trends. Spatially, the limited number of observation sites constrains characterization of regional patterns. Differences in sampling and analytical methods also limit direct comparisons among sites. In terms of composition, although Section 3.2 summarizes the characteristics of aliphatic, aromatic, oxygenated, and other non-hydrocarbon components, a substantial fraction of S/IVOCs remains unresolved as UCM, precluding complete molecular-level identification and quantification.

## 4. Contribution of S/IVOCs to SOA Formation

Secondary organic aerosol (SOA) is formed through the atmospheric oxidation of organic precursors. In this review, ambient SOA concentration refers to SOA present in atmospheric particles, which can be estimated from aerosol composition measurements. SOA formation potential (SOAFP) represents the potential SOA production from measured precursors based on aerosol formation coefficients or assumed oxidation conditions and yields. Other precursor-based estimates may additionally consider reaction kinetics and photochemical exposure. Model-derived SOA contributions depend on emission inventories, chemical mechanisms, and model parameterizations. Therefore, these quantities should be distinguished when comparing results across studies.

To further clarify the comparability of these quantities, the principal SOA assessment metrics used in the reviewed studies are summarized in Table 4 according to precursor coverage, oxidation conditions, key assumptions, and major sources of uncertainty.

### 4.1. Ambient-Measurement-Based SOA Formation Estimates

Given the pronounced variations in S/IVOC concentrations and composition across different atmospheric environments, SOA formation estimates derived from ambient precursor measurements may also vary among regions and source-influenced settings. Multiple field studies have combined measured atmospheric S/IVOC concentrations with reaction-rate, oxidation-exposure, and/or SOA-yield parameters to estimate potential SOA formation. These calculations provide useful comparisons of precursor importance within the assumptions and compound coverage of each study, but they should not be interpreted as direct source-apportioned contributions to observed ambient SOA. Zhang et al. [44] measured S/IVOCs in Beijing during autumn 2020 and estimated SOA formation using a bottom-up yield method based on measured precursor concentrations, OH reaction-rate constants, assumed OH exposure, and SOA yields. The estimated SOA mass associated with S/IVOCs was 0.70 ± 0.57 μg m^−3^, comparable to the 0.62 ± 0.61 μg m^−3^ estimated for the measured VOCs. Within the precursor set included in that calculation, S/IVOCs accounted for approximately 53% of the total estimated SOA, despite their measured mass being only about 5% of the measured VOC mass. Zhou et al. [24] estimated SOA formation from measured gas-phase VOC and S/IVOC precursors in Chengdu using a bottom-up yield method that incorporated precursor concentrations, OH-reaction kinetics, photochemical age, and chamber-derived SOA yields. Within this calculation, gas-phase S/IVOCs accounted for 17.2% of the measured gas-phase organic mass and were associated with 0.18 ± 0.23 μg m^−3^ of estimated SOA, corresponding to 27.4% of the total estimated gas-phase SOA production from the included precursors. Li et al. [36] found that oxygenated PAHs (Oxy-PAHs) accounted for 8.3% of the total S/IVOC concentration in Shenzhen in winter 2023 but contributed approximately 19% of the estimated SOA derived from the measured S/IVOCs, further illustrating the importance of S/IVOCs in SOA formation.

Compared with the urban measurement-based estimates described above, relatively high IVOC SOA formation potentials were reported for petrochemical and riverside industrial environments. Based on measured IVOC data and yield parameters, Zhang et al. [33] estimated an overall SOA formation potential of 11.45 μg m^−3^ for gas-phase IVOCs in the studied petrochemical area. Hou et al. [32] reported calculated IVOC SOA formation potentials of 80.9 ± 36.5, 79.2 ± 20.5, and 63.4 ± 15.2 μg m^−3^ for the morning, afternoon, and evening, respectively, during summer, compared with 13.4 ± 2.6, 13.2 ± 2.7, and 12.6 ± 1.9 μg m^−3^ during winter. According to the supplementary information accompanying Hou et al. [32], the SOA formation potentials were calculated for a specified 48-h photo-oxidation scenario using an OH concentration of 1.5 × 10^6^ molecules cm^−3^ and literature-based reaction-rate constants and SOA yields under high-NO_x_ conditions at an organic-aerosol concentration of 20 μg m^−3^. The substantially higher summer values coincided with much higher IVOC concentrations during summer than during winter and therefore reflect both elevated precursor abundance and the assumed oxidation and yield conditions.

Compared with urban and industrial environments, quantitative studies of the contribution of S/IVOCs to SOA in regional background and forest environments remain limited and have mainly focused on specific biogenic components [38].

### 4.2. Laboratory-Based Assessments

Vehicle dynamometer studies have quantified the emission composition of S/IVOCs and estimated substantial associated SOA formation. Zhao et al. [10] tested diesel vehicles on a dynamometer and found that more than 90% of IVOCs were emitted as a UCM. By combining measured emissions with previously reported SOA yields and reaction-rate parameters, they estimated that IVOCs accounted for approximately 99% of the predicted SOA from the included precursors; under complete oxidation, the estimated SOA production from IVOCs substantially exceeded directly emitted POA. For gasoline vehicles, Drozd et al. [45] tested 20 in-use light-duty gasoline vehicles meeting different emission standards on a chassis dynamometer and estimated their SOA formation potential using measured emissions and literature SOA yields. Under a 24 h oxidation scenario, IVOCs contributed 45% of the estimated SOA from gasoline-vehicle exhaust, indicating that IVOCs are important SOA precursors from gasoline vehicles and should be considered in mobile-source emission inventories and SOA models.

Laboratory simulations of combustion sources indicate that S/IVOCs emitted from residential coal and biomass combustion can make substantial contributions to SOA formation. Using a laminar-flow oxidation reactor, Li et al. [12] estimated that IVOCs from wood-burning emissions contributed approximately 70% of the SOA formed in the reactor, more than twice the contribution attributed to VOCs. Cai et al. [57] reported similar results for household coal combustion in northern China. For widely used bituminous coal, S/IVOCs accounted for only approximately 30% of total gaseous non-methane organic compound (NMOC) emissions, yet under complete photo-oxidation, they contributed more than 70% of the SOA formation potential from coal-combustion gaseous organics. Thus, neglecting S/IVOCs may substantially underestimate the SOA formation potential of household bituminous-coal combustion.

Robinson et al. [4] challenged the conventional assumption that primary organic aerosol is nonvolatile and chemically inert, proposing a dynamic framework for organic aerosol evolution. During dilution to ambient concentrations, primary organic emissions can undergo gas–particle repartitioning; some particle-phase SVOCs evaporate into the gas phase and, together with primary IVOCs, undergo photochemical oxidation. The resulting decrease in volatility allows oxidation products to repartition into the particle phase and form SOA. Subsequent smog-chamber studies examined the effects of molecular structure, NO_x_ conditions, and gas–particle partitioning on SOA formation from S/IVOC oxidation. Chan et al. [46] studied the gas-phase photo-oxidation of naphthalene, methylnaphthalenes, and dimethylnaphthalenes and measured SOA yields of 0.19–0.45 under high-NO_x_ conditions. Under low-NO_x_ conditions, the yields of naphthalene, 1-methylnaphthalene, and 2-methylnaphthalene were 0.73, 0.68, and 0.58, respectively. The authors attributed the high-NO_x_ results mainly to ring-opening products and semi-volatile SOA, whereas low-NO_x_ oxidation generated more ring-retaining, low-volatility products and consequently higher and relatively stable SOA yields. Presto et al. [47] used n-heptadecane as a representative compound and found that multigenerational photo-oxidation under high-NO_x_ and low-organic-aerosol conditions could form highly oxidized SOA. These results suggest that multigenerational oxidation of IVOCs may be an important pathway for ambient oxidized organic aerosol formation, though the process is strongly influenced by NO_x_ levels, OH exposure, and gas–particle partitioning.

### 4.3. Model-Based Assessments

Incorporating S/IVOC emissions into air-quality models has improved SOA simulations in several studies. Wu et al. [48] used WRF-Chem to simulate a winter pollution episode in China from 21 to 31 December 2017. After including S/IVOC emissions and degradation processes, the mean bias in simulated SOA decreased by 25%, and the fraction of observed SOA reproduced by the model increased from 18% to 40%. Modeling studies by Li et al. [49,56] in the Beijing–Tianjin–Hebei region likewise showed that including primary S/IVOC emissions and chemical aging improved the reproduction of SOA growth during severe haze episodes. In the Pearl River Delta, Wu et al. [42] found that incorporating an S/IVOC emission inventory into WRF-Chem increased the simulated regional mean SOA concentration by 161% relative to the simulation without S/IVOC emissions, substantially reducing the gap between modeled and observed concentrations.

Different modeling frameworks and parameterization schemes produce different estimates of S/IVOC contributions to SOA. Huang et al. [50] used CAMx to investigate SOA formation over the Yangtze River Delta in July 2018 and found that introducing IVOC emissions increased regional SOA concentrations by approximately 5–26%. Wang [51] further reported that replacing the default SOAP mechanism in CAMx with a 1.5D-VBS mechanism increased the monthly mean simulated SOA concentration at the Dianshan Lake site by 75.6% in July 2018. Yao et al. [52] introduced 1D- and 1.5D-VBS mechanisms into CAMx for a year-long simulation of SOA over the Pearl River Delta from April 2011 to March 2012. Both VBS schemes reduced, to some extent, the underestimation of SOA by the conventional model, with the 1D-VBS showing better overall performance. Relative to the conventional SOA scheme, incorporating IVOC/SVOC evaporation, oxidation, and gas–particle partitioning in the 1D-VBS increased the simulated annual mean SOA concentration by approximately 4–7 μg m^−3^ in central and surrounding areas of the Pearl River Delta, corresponding to an increase of approximately 400–500%. Zhao et al. [53] compared three SOA parameterizations: pcVOC (potential VOC from combustion emissions, scaled from POA emissions using a fixed factor), IVOC-VBS, and 2D-VBS. Model evaluation against observations from multiple sites in China showed that total SOA simulated by the 2D-VBS scheme had the best overall agreement with observations, although substantial deviations remained in some regions.

Uncertainties in aging mechanisms and emission inventories are major sources of bias in current model simulations. Oak et al. [54] reported that including S/IVOCs and chemical aging improved surface SOA simulations in Seoul, but most schemes still underestimated the increase in SOA with photochemical age aloft within the boundary layer, indicating that aging processes at higher altitudes remain insufficiently represented. Modeling by Giani et al. [55] over the Po Valley in Italy also showed that lower wintertime photochemical activity reduced model sensitivity to aging mechanisms, making the accuracy of S/IVOC emissions in the inventory particularly important under these conditions.

### 4.4. Limitations and Uncertainties in Current SOA Formation Assessments

Field observations, laboratory experiments, and numerical simulations collectively indicate that S/IVOCs can make important contributions to SOA formation, but substantial uncertainty remains in accurately quantifying the magnitude of this contribution.

For observation-based SOA estimates, results are strongly influenced by the range of species included. Different studies cover different precursor sets and use different SOA-yield parameters, making the resulting contribution estimates difficult to compare directly.

SOA yields measured in laboratory experiments are not necessarily directly transferable to the ambient atmosphere. Most yield parameters are derived from smog-chamber oxidation experiments using individual compounds, whereas the atmosphere contains complex mixtures of numerous organic species that can undergo interacting chemical processes. Laboratory studies also cannot fully reproduce the coexistence of multiple precursors or precisely capture the effects of variations in light intensity, humidity, and aerosol phase state on gas-particle partitioning. Consequently, applying chamber-derived yield parameters directly to field-based contribution estimates can introduce uncertainty associated with differences between experimental and ambient conditions.

Uncertainties in numerical simulations arise mainly from emission inventories and parameterization schemes. Different VBS formulations (1D-VBS, 1.5D-VBS, and 2D-VBS) can produce substantially different results, and the representation of chemical aging also affects SOA estimates. Many existing VBS frameworks draw heavily on studies of traffic emissions, whereas the contribution of nontraditional urban sources such as VCPs is increasing; therefore, applying legacy parameterizations may not adequately represent the current contribution of S/IVOCs.

## 5. Human Health Risks of S/IVOCs: Current Evidence and Limitations

Direct toxicological evidence for atmospheric S/IVOCs as a complete chemical mixture remains limited. Most quantitative health-risk evidence relevant to the S/IVOC volatility range is currently compound-specific and is concentrated on relatively well-characterized classes such as PAHs and related polycyclic aromatic compounds (PACs). These compounds represent only a subset of the chemically diverse S/IVOC mixture, and their toxicity should therefore not be extrapolated to unresolved S/IVOCs or to the entire S/IVOC class. For example, Chen et al. [58] conducted a probabilistic risk assessment specifically for PAHs measured in industrial, traffic, and rural environments in Taiwan during 2002–2003. For adults, the geometric mean incremental lifetime cancer risk was 1.04 × 10^−4^ for inhalation and 3.85 × 10^−5^ for dermal contact. Wei [59] similarly evaluated PACs in both ambient air and road dust in Xi’an. The airborne PAC results are directly relevant to atmospheric exposure, whereas ingestion and dermal-contact risks estimated for road dust represent exposure through a separate environmental medium rather than direct exposure to airborne S/IVOCs. Although atmospheric deposition may contribute to the transfer of particle-associated organics to surfaces, the cited evidence does not establish atmospheric deposition as the exclusive source of the road-dust PACs, and these pathways are therefore not treated here as direct atmospheric exposure routes. These studies provide health-risk evidence for selected PAHs/PACs rather than direct toxicological evidence for the complete atmospheric S/IVOC mixture.

Beyond PAHs/PACs, other classes of S/IVOCs, including phthalate esters, organophosphate flame retardants (OPFRs), polybrominated diphenyl ethers, and alternative plasticizers, also warrant consideration. Xiang et al. [60] investigated these compounds in ambient air near a petrochemical industrial area and assessed the inhalation-related cancer risks of PAHs and di(2-ethylhexyl) phthalate (DEHP). The estimated lifetime lung cancer risk associated with PAHs was moderate, whereas the incremental lifetime cancer risk for DEHP was below the acceptable level adopted in that study. These findings indicate that health risks may differ among individual S/IVOC classes and highlight the need for compound-specific assessments beyond PAHs.

Experimental studies have identified several toxicological mechanisms for specific S/IVOCs, including oxidative stress, cytotoxicity, genotoxicity, and neurotoxicity. Oxidative stress, involving excessive reactive oxygen species (ROS) production and impaired antioxidant defenses, can contribute to cellular injury and inflammation. You et al. [61] found that DEHP co-exposure with ovalbumin increased ROS and malondialdehyde levels and reduced glutathione in a mouse asthma model, while vitamin E attenuated these changes and associated airway responses. Regarding acute and short-term toxic effects, Meng et al. [62] reported that 72-h exposure to selected OPFRs reduced cell viability and induced oxidative stress, intracellular calcium accumulation, and C/EBP homologous protein/caspase-3-related apoptosis in human lung cancer cells. Costa et al. [63] also observed oxidative stress and apoptotic responses in mice within 24 h following a single oral exposure to 2,2′,4,4′-tetrabromodiphenyl ether (BDE-47). For carcinogenic pathways, Vázquez-Gómez et al. [64] demonstrated that benzo[a]pyrene activated Aryl hydrocarbon receptor/Src/Extracellular signal-regulated kinase signaling and promoted cytochrome P450 1A1 expression, contributing to metabolic activation and DNA adduct formation in human bronchial epithelial cells and providing mechanistic evidence of genotoxic damage. Regarding neurotoxicity, Costa et al. [63] further showed that BDE-47 induced oxidative stress and neuronal apoptosis in cultured mouse cerebellar neurons and exposed mice. These findings help explain potential toxicological effects of selected S/IVOCs, but differences in experimental doses, exposure routes, and study models limit their direct extrapolation to human health risks under typical atmospheric exposure conditions.

Indoor exposure studies provide additional evidence regarding the potential health effects of specific S/IVOCs. Bornehag et al. [65] reported associations between DEHP in household dust and childhood asthma, while butyl benzyl phthalate was associated with rhinitis and eczema. Similarly, Meeker and Stapleton [66] found that OPFRs in household dust were associated with changes in reproductive and thyroid hormone levels and reduced sperm concentration in men. Although these observational findings do not directly represent outdoor inhalation exposure, they provide complementary evidence of potential health effects associated with SVOCs.

Atmospheric transformation may alter the toxicological properties of S/IVOCs, but current evidence is insufficient to quantify how oxidation changes the health risk of the overall atmospheric S/IVOC mixture. Yan et al. [19] summarized potential health concerns associated with IVOCs and their oxidation products, while substantial uncertainty remains because of the complex composition of IVOCs and the limited availability of toxicity data for many oxidation products and unresolved compounds. Therefore, the potential toxicity of atmospheric oxidation products is still an important research gap.

Current health-risk assessments relevant to atmospheric S/IVOCs are constrained primarily by limited toxicological and exposure data. Toxicity reference values are available for only a small subset of identified compounds, largely selected PAHs and phthalates, whereas many IVOCs, unresolved mixture components, and atmospheric oxidation products lack reliable toxicological parameters. Existing quantitative assessments therefore characterize only the risks associated with the compounds explicitly included in each study and cannot be considered representative of the complete atmospheric S/IVOC mixture. In addition, the toxicological effects of combined exposure to multiple unresolved compounds remain poorly constrained. Accordingly, current evidence is insufficient to quantify the overall health risk of atmospheric S/IVOCs, and future assessments require broader compound identification, toxicological characterization, and mixture-level toxicity studies. For atmospheric S/IVOCs, inhalation is the most direct exposure pathway considered in this review, whereas ingestion and dermal contact are discussed only when atmospheric organics are measured in or transferred to other environmental media such as deposited dust or surfaces; such pathways should be distinguished from direct airborne exposure.

## 6. Conclusions and Perspectives

Taken together, the central challenge emerging from current S/IVOC research is the propagation of uncertainty from chemical measurement and ambient characterization to SOA assessment and health-risk evaluation.

Regarding analytical methods, current S/IVOC measurements have developed into complementary online-monitoring and offline-analysis frameworks. Online methods allow real-time sample introduction at the observation site, whereas offline GC–MS and GC×GC–MS offer advantages for separating complex mixtures, non-target screening, and chemical-class characterization. Nevertheless, adsorption and volatilization losses during sampling, compositional biases introduced by thermal desorption or solvent extraction, limited availability of authentic standards, and compound-dependent instrumental responses remain major constraints on the comprehensive quantification of S/IVOCs and cross-study comparability. Future work should strengthen standardization of sampling, calibration, and quality-control procedures, conduct intercomparisons among analytical platforms, and combine online and offline methods to improve characterization of unknown and unresolved components.

Regarding pollution characteristics, existing studies show that S/IVOC concentrations, composition, and temporal variability depend on site type and the spatial and temporal scope of measurement. Elevated concentrations can occur in environments strongly influenced by industrial activities, traffic, and combustion, but species coverage and analytical methods differ among studies. Seasonal and diurnal patterns are likewise not uniform and are jointly influenced by emission activity, atmospheric transport, boundary-layer evolution, gas-particle partitioning, and photochemical transformation. Most field studies are single-site campaigns lasting only several weeks to months, and therefore have limited spatial coverage and long-term representativeness. Future studies should establish coordinated long-term observation networks covering different site types and seasons and should standardize reporting of target compounds, phase coverage, quantification methods, and quality-control information to support regional comparisons, emission-inventory evaluation, and long-term trend analysis.

Regarding the contribution of S/IVOCs to SOA formation, field observations, source-emission tests, smog-chamber experiments, and numerical simulations collectively indicate that S/IVOCs can act as important SOA precursors in diverse environments; under some urban, industrial, and combustion-source conditions, estimated SOA formation from S/IVOCs can be comparable to or greater than that from conventional VOCs. However, different studies use different approaches to evaluate S/IVOC contributions to SOA, including contribution estimates based on different parameter settings, SOA formation potential calculations, and numerical simulations, and the resulting values cannot be compared directly. Estimates are also influenced by precursor coverage, yield parameters, oxidation time, NO*_x_* levels, organic aerosol loading, and gas–particle partitioning. Incorporating S/IVOC emissions and aging processes into air-quality models can improve SOA simulations for specific pollution episodes, although substantial uncertainties remain in emission inventories, volatility distributions, and multigenerational oxidation parameterizations. As experimental and observational datasets continue to expand, machine learning may be explored as a means of simplifying simulations of complex S/IVOC oxidation and SOA formation and improving computational efficiency; however, the applicability and reliability of such models still require validation against laboratory experiments and field observations.

Regarding health effects, current evidence is concentrated on specific S/IVOCs, such as PAHs, for which toxicity parameters are relatively well established, whereas many IVOCs, unresolved mixtures, and their oxidation products still lack reliable exposure and toxicological data. Current risk assessments therefore reflect only a limited number of known compounds and cannot represent the health risks of the entire S/IVOC mixture. Predictions of toxicity parameters and assumptions used to define exposure scenarios also affect the results, and the direction and magnitude of the resulting uncertainty are not fully constrained. Future studies should integrate non-target analysis with multipathway exposure models, expand toxicity testing of unidentified S/IVOCs and their oxidation products, and strengthen research on susceptible populations such as children and on combined exposure effects. Integrating emission sources, atmospheric transformation, human exposure, and toxic effects within a unified framework will help identify compounds with both high SOA formation potential and potential health hazards.

## Figures and Tables

**Table 1:** Online analytical methods for S/IVOCs.

Method	Principle	Advantages	Limitations	Analytes	References
In situ automated GC–MS	On-site automated sampling–multisorbent trap preconcentration–TD-GC–MS	Suitable for long-term unattended operation at field sites	Complex quantification procedure	VOCs/IVOCs (including halogenated and oxygen-/nitrogen-containing compounds) up to C_15_; atmospheric organic compound identification	[21]
Thermal desorption aerosol gas chromatograph (TAG)	Particles are humidified and collected by inertial impaction, followed by thermal desorption into GC–MS	Measures semi-volatile and nonvolatile particle-phase organic compounds	Relatively low temporal resolution (hourly); thermally labile compounds may decompose	Semi-volatile and nonvolatile particle-phase organic compounds	[22]
Semi-volatile thermal desorption aerosol gas chromatograph (SV-TAG)	High-surface-area stainless-steel fiber filter; gaseous organics adsorb onto the filter surface and particles are collected by filtration	Extends coverage to relatively volatile gas-phase IVOCs and particle-phase SVOCs	Relatively low temporal resolution (hourly); thermally labile compounds may decompose	Relatively volatile gas-phase IVOCs and particle-phase SVOCs	[14]
Online thermal desorption GC–MS (online TD-GC–MS)	Automated preconcentration–cryofocusing–TD-GC–MS	High degree of automation; optimized for IVOCs (e.g., reduced wall losses); relatively stable quantification	High maintenance cost; potential carryover and blockage for IVOCs	IVOCs (alkanes, PAHs, and UCM)	[23]
Online comprehensive two-dimensional gas chromatography–mass spectrometry (online GC×GC–MS/GC×GC-ToF-MS)	Two-dimensional separation using two columns in series	High peak capacity; resolves UCM chemical groups	Complex data processing	Common S/IVOCs; UCM	[15,27,28]
High-resolution time-of-flight chemical ionization mass spectrometry (HR-ToF-CIMS)	Chemical ionization through reagent-ion–analyte reactions; detectable compound range and sensitivity depend on reagent-ion chemistry	High temporal resolution	Reagent-ion-dependent compound coverage and sensitivity; difficult isomer discrimination; challenging quantification	HOMs and polar oxidation products	[27,28]
Extractive electrospray ionization time-of-flight mass spectrometry (EESI-ToF-MS)	Extraction of aerosol particles by electrospray droplets followed by ionization	Nonthermal online analysis that avoids thermal decomposition during conventional thermal desorption	Molecular-formula information does not establish molecular structure or isomer identity; quantification is less robust and depends on calibration standards	Particle-phase organic compounds	[28]

**Table 2:** Offline analytical methods for S/IVOCs.

Method	Principle	Advantages	Limitations	Analytes	References
Filter–adsorbent sampling + solvent extraction + GC–MS	A quartz-fiber filter in series with PUF/XAD-2 separately collects particle- and gas-phase S/IVOCs; solvent extraction and concentration are followed by GC–MS qualitative and quantitative analysis	Mature methodology; reliable qualitative and quantitative analysis; physical separation of gas and particle phases; samples are readily stored	Labor-intensive pretreatment and high solvent consumption; sampling artifacts can cause positive/negative bias (gas adsorption on filters and volatilization under airflow)	n-Alkanes, PAHs, phthalates, etc.	[20,29,30]
Sorbent-tube sampling + thermal desorption + GC–MS or GC×GC–MS (offline TD-GC–MS/GC×GC–MS)	Gas-phase samples are collected directly on sorbent tubes, thermally desorbed, and introduced into GC–MS or GC×GC–MS	No organic solvent required; environmentally favorable; suitable for gas-phase SVOC/IVOC analysis	Sample is consumed in a single analysis; repeated injection is not possible; requires precise operation and stable instrumentation; 1D-GC–MS has limited ability to resolve UCM isomers	n-Alkanes, PAHs, alkylcyclohexanes, etc.	[20,26,29,30]
Filter/adsorbent sampling + solvent extraction/thermal desorption + GC×GC–MS/GC×GC-ToF-MS non-target analysis	Quartz-fiber filters and adsorbents separately collect particle- and gas-phase S/IVOCs; filter samples are solvent-extracted, whereas adsorbent samples are solvent-extracted or thermally desorbed before comprehensive two-dimensional gas chromatographic analysis.	High peak capacity; suitable for non-target screening and chemical-class characterization of complex S/IVOCs	High instrument and maintenance costs	Complex S/IVOCs; UCM	[20]

**Table 3:** Gas- and particle-phase concentrations of atmospheric IVOCs and S/IVOCs at different observation sites.

Site Type	Location/Period	Analytical Methods and Compound Ranges	Gas-Phase Concentration (μg m^−3^)	Particle-Phase Concentration (μg m^−3^)	Gas + Particle-Phase Concentration (μg m^−3^)	Reference
Near-source/industrial area	Yangzhou riverside petrochemical industrial park, China/summer 2024	TD–GC–MS; C_12_–C_22_ n-alkanes, 8 PAHs, branched alkanes, UCM (B_12_–B_22_)	-	-	172 ± 25.0 *	[32]
Near-source/industrial area	Yangzhou riverside petrochemical industrial park, China/winter 2025	TD–GC–MS; C_12_–C_22_ n-alkanes, 8 PAHs, branched alkanes, UCM (B_12_–B_22_)	-	-	31.8 ± 2.45 *	[32]
Near-source/industrial area	Typical petrochemical industrial area, China/summer 2021	ASE–2D-GC–MS/FID; C_12_–C_35_ n-alkanes, 16 PAHs, O/N-containing organics and UCM (B_10_–B_35_ bins)	108.87 ± 78.93 *	4.22 ± 1.54 *	-	[33]
Near-source/industrial area	Shanghai Yangshan Port, China/summer 2016 and winter 2017	TD–GC–ToF–MS; C_12_–C_22_ n-alkanes, 8 PAHs, branched alkanes and UCM	5.1 ± 0.8 *	-	-	[34]
Urban area	Urban Shanghai, China/winter 2016	TD–GC–MS; C_12_–C_22_ n-alkanes, PAHs, UCM (B_12_–B_22_)	-	-	58.5 ± 27.0 *	[35]
Urban area	Urban Shanghai, China/summer 2017	TD–GC–MS; C_12_–C_22_ n-alkanes, PAHs, UCM (B_12_–B_22_)	-	-	6.8 ± 3.7 *	[35]
Urban area	Shenzhen, China/winter 2023	TD–GC×GC–MS; C_9_–C_33_ n-alkanes and 10 chemical classes, including acids, ketones and Oxy-PAHs	9.87 ± 5.92 **	-	-	[36]
Urban area	Chengdu, China/winter 2021	TD–GC×GC–qMS; 351 compounds, including C_7_–C_33_ n-alkanes, acids, ketones, esters and PAHs	2.63 ± 1.25 **	1.83 ± 1.15 **	-	[24]
Urban area	Guangzhou, China/autumn 2019	TD–GC–MSD; n-alkanes, PAHs, branched alkanes and residual UCM (B_12_–B_22_ bins)	25.0 ± 0.95 *	-	-	[37]
Urban area	Los Angeles, USA/summer 2010	TD–GC–MS; 20 speciated IVOCs and UCM (C_12_–C_22_ n-alkane-equivalent volatility bins)	6.3 ± 1.9 *	-	-	[8]
Urban area	London, UK/winter–spring 2017	TD–GC×GC–ToF–MS; C_13_–C_36_ linear/branched alkanes, C_12_–C_25_ monocyclic alkanes, C_13_–C_27_ bicyclic alkanes and C_10_–C_24_ monocyclic aromatics	-	-	0.587–2.448 **	[25]
Background area	Amazon, Brazil/wet season 2014	SV-TAG–GC–MS; 30 sesquiterpenes (C_15_), 4 diterpenes (C_20_)	0.0418 **	-	-	[38]
Background area	Amazon, Brazil/dry season 2014	SV-TAG–GC–MS; 30 sesquiterpenes (C_15_), 4 diterpenes (C_20_)	0.0388 **	-	-	[38]
Background area	Colorado, USA/summer 2011	SV-TAG–AMS; hydrocarbon-like compounds in the C_14_–C_26_ n-alkane-equivalent volatility range	0.7 **	-	-	[14]

Note: “-” indicates no data available; “*” indicates IVOC concentration; “**” indicates S/IVOC concentration. Concentrations in the table are mean values or ranges obtained at corresponding study sites (single or multiple sites) during the sampling periods.

**Table 4:** Comparison of major metrics used to assess SOA formation from S/IVOCs.

Metric	Precursor Coverage	Oxidation Timescale/Conditions	Key Assumptions	Main Uncertainty
Ambient-measurement-based SOA estimate [24,36,44]	Measured ambient VOCs and/or S/IVOCs included by the analytical method	Study-specific photochemical age or prescribed OH exposure; literature reaction rates and SOA yields	Measured precursor concentrations and assigned kinetic/yield parameters approximate SOA formation from the included precursor set	Incomplete precursor coverage; unresolved compounds; uncertain yields or photochemical age; omission of some multiphase processes
SOA formation potential (SOAFP) [32,33,45]	Measured ambient concentrations or source-emission S/IVOCs included in the calculation	Prescribed oxidation scenario (e.g., 24 h [45] or 48 h [32]) or complete-oxidation assumption	Literature reaction-rate constants and SOA yields are transferable to the measured or emitted precursor mixture under the specified scenario	Strong dependence on OH exposure, oxidation time, NO_x_ regime, organic-aerosol loading, and yield assignment
Chamber-/reactor-derived SOA yield [12,46,47]	Selected individual S/IVOCs or source-emission mixtures tested under controlled conditions	Experiment-specific oxidant exposure, NO_x_ level, aerosol loading, dilution, and reaction time	Controlled experiments reproduce the relevant chemistry of the tested precursor or source mixture	Wall losses; limited representation of ambient dilution, humidity, mixed precursors, aging, and gas-particle partitioning
Model-derived SOA change or contribution [42,47,48,49,50,51,52,53,54,55,56]	Inventory-defined S/IVOC emissions represented by model surrogates or volatility bins	Multi-day to annual simulations with model-specific chemistry, aging, and gas-particle partitioning	Emission inventories, volatility distributions, chemical mechanisms, and VBS parameterizations adequately represent atmospheric S/IVOC evolution	Emission-inventory and source-coverage uncertainty; VBS formulation; aging and partitioning parameterization; meteorology

## Data Availability

No new data were created or analyzed in this study. Data sharing is not applicable to this article.
